# The spectrum of pediatric amplified musculoskeletal pain syndrome

**DOI:** 10.1186/s12969-020-00473-2

**Published:** 2020-10-12

**Authors:** David D. Sherry, Maitry Sonagra, Sabrina Gmuca

**Affiliations:** 1 Department of Pediatrics, Division of Rheumatology, Children’s Hospital of Philadelphia, 3501 Civic Center Blvd, Philadelphia, PA 19104-3820 USA; 2 University of Pennsylvania Perelman School of Medicine and Children’s Hospital of Philadelphia, Philadelphia, PA USA; 3 Center for Pediatric Clinical Effectiveness, Children’s Hospital of Philadelphia, Roberts Center for Pediatric Research, Philadelphia, PA 19146 USA; 4 Policy Lab, Children’s Hospital of Philadelphia, Roberts Center for Pediatric Research, Philadelphia, PA 19104 USA

**Keywords:** Pain, Amplified pain, Fibromyalgia, Widespread pain, Limited pain, Intermittent pain, Child, Adolescent

## Abstract

**Background:**

Children presenting with musculoskeletal pain to pediatric rheumatology clinics are very heterogeneous and on a continuum from those with localized pain to total body pain. Many report intermittent, rather than constant, pain. We examined clinical and psychological characteristics of these children at presentation and specifically those who fulfilled the criteria for fibromyalgia.

**Methods:**

We performed a retrospective, cross-sectional cohort study of children under ≤18 years old presenting to the pediatric rheumatology pain clinic between January 2015 and July 2019 and enrolled in a patient registry. We included children diagnosed with amplified pain, excluding those fulfilling criteria for complex regional pain syndrome. Abstracted data included clinical characteristics, pain symptoms, functional disability inventory (FDI), widespread pain index, and symptom severity scale.

**Results:**

We analyzed 636 subjects, predominantly non-Hispanic Caucasian females. Using median split method, 54% had diffuse pain (≥ 5 body regions involved), but, of these, only 58% met criteria for fibromyalgia. Subjects with diffuse pain, compared to those with localized pain had a longer duration of pain (24 vs 12 months, *p* < 0.01), reported greater pain intensity (6/10 vs 5/10, *p* < 0.001), greater mental health burden, and poorer function (FDI 25 vs 19, *p* < 0.0001). Subjects with limited pain more often reported a history of trigger event (34% vs 24%, *p* < 0.01) but not autonomic changes (14% vs 14%, *p* = 0.94). The presence of adverse childhood experiences did not differ among those with limited versus diffuse pain except for parental divorce (16% vs 23%, *p* = 0.03). Intermittent pain was reported in 117 children (18%) and, compared to subjects with constant pain, they reported less pain (0/10 vs 6/10) and were more functional (FDI 13 vs 25) (both *p* < 0.0001).

**Conclusions:**

There exists a wide spectrum of pain manifestations among children with amplified pain including limited or diffuse and constant or intermittent pain. Most children who presented to our clinic did not fulfill criteria for fibromyalgia but nonetheless had significant symptoms and disability. Studies focusing on fibromyalgia may miss the full extent of childhood amplified pain. Additionally, research limited to those meeting the fibromyalgia criteria likely underestimate the significant impact of amplified pain among the pediatric population.

Pediatric chronic non-inflammatory musculoskeletal pain gained widespread medical attention in the middle of the twentieth century [1]. The nascency of the field resulted in a host of names for the presentations of idiopathic musculoskeletal pain [2–6]. Amplified musculoskeletal pain syndrome (AMPS) is a term that encompasses the spectrum of manifestations of chronic pediatric musculoskeletal pain. The common thread underlying these different subtypes is central and/or peripheral sensory pain amplification, hence the name amplified musculoskeletal pain [7]. The term AMPS is understandable and provides the patient with a mechanism by which to understand and validate the reality of his/her pain.

Most reports focus on one specific subset of chronic pain such as fibromyalgia, widespread pain, idiopathic localized or diffuse pain, functional pain (frequently functional abdominal pain), or headache [2, 8–10]. One group uses complex regional pain syndrome (CRPS) spectrum disorder to include patients with limited pain but who did not fulfill CRPS criteria [11]. However, we argue that these presentations are subsets of pediatric amplified pain. Many children exhibit features of multiple subsets of AMPS, having one initial subset followed later by another, or develop intermittent symptoms [12]. Those with intermittent pain are not well described in the literature and frequently languish without a diagnosis. It does not seem reasonable that these children have multiple different pain conditions, but rather one condition with varying manifestations that may fluctuate in an individual over time. An appreciation of where these children fit into the spectrum may lead to insights into the pathophysiology and psychopathology of the condition in addition to having implications for treatment.

The purposes of this study are 1) to further define the presenting characteristics of children with chronic non-inflammatory musculoskeletal pain to a pediatric rheumatology academic center and 2) determine whether there are significant differences in presenting manifestations, especially in those who fulfill the criteria for fibromyalgia, those with more limited areas of pain versus those with diffuse pain, and those with intermittent pain versus constant pain. First, we hypothesized that children with diffuse pain would generally fulfill adult criteria for fibromyalgia, be more functionally disabled and experience a longer duration of pain than those with limited pain. Second, we hypothesized children with limited pain would manifest more transient autonomic changes, a *forme fruste* of CRPS. Third, we hypothesized that children with intermittent pain would typically be those with more limited pain, would have a higher level of function, and would have a longer duration of pain than those with constant pain since they are frequently pain free when seen and are not well described in the literature. Insights gained from addressing these aims may improve the diagnosis and treatment of children with chronic musculoskeletal pain, thereby mitigating the associated excessive socioeconomic and psychological costs.

## Methods

### Study site and participants

This was a retrospective cross-sectional cohort study of subjects ≤18 years old diagnosed with AMPS between January 2015 and July 2019 enrolled in an IRB approved patient registry. This prospective patient registry captures data from patients’ initial clinic visit as well as all subsequent follow-up visits and included approximately 96% all new patients. AMPS was defined as pain disproportionate to the stimulus without other medical explanation such as inflammation [12]. The treating pediatric rheumatologist diagnosed AMPS on the basis of physical examination, patient history, and the absence of an underlying condition explaining pain. Juvenile fibromyalgia was defined according to the 2010 criteria of the American College of Rheumatology for fibromyalgia in adults [13]. We excluded children with prominent and prolonged autonomic changes seen in CRPS, such as cyanosis, coolness to the extremity, edema or perspiration changes [14]. We included subjects who reported fleeting hot or cold changes, swelling, or other symptoms that were not evidenced on examination and did not fulfill criteria for CRPS [11]. Although CRPS can be included in the entire spectrum of children with chronic non-inflammatory pain, CRPS is generally easily diagnosed. The focus of this study was to concentrate on those who are more difficult to diagnose and who represent a much larger proportion of children presenting with chronic pain.

### Clinical characteristics

Patient data included demographics, family history, medical, surgical, and psychiatric history; physical examination findings; patient reported outcomes (PROs); and medical interventions. Self-reported psychological variables and PROs including the Functional Disability Inventory (FDI) [15, 16], verbal pain score (0–10), widespread pain index (WPI; range 0–19), and symptom severity score (SSS; range 0–12) were collected via standardized intake forms and reviewed during the clinic visit [15, 16]. Registry data captured patient reported adverse childhood experiences (ACEs) abstracted from the patient’s medical record or psychologist’s clinical evaluation note. ACEs were defined as any potentially traumatic events that happened to the patient during childhood, which can have negative, lasting effects on health and well-being of the child [17]. We included history of the following as an ACE: verbal abuse, physical abuse, sexual abuse, parent with an alcohol problem, parent with a drug problem, parents are divorced or separated, other household member with drug or alcohol problem, household member has attempted or committed suicide or had been incarcerated, history of economic hardship in the family (e.g. not having enough money for food or clothing), patient’s mother or stepmother a victim of domestic violence or any other negative experience such as bullying [18]. All the data from the subjects’ medical records were abstracted into the secure Research Electronic Data Capture (REDCap) database system to create a registry database. We a priori categorized body pain into 13 different regions: head, neck, face, right hand, left hand, back/flanks, chest, abdomen, right leg/hip, left leg/hip, buttocks/pelvis/groin, fingers/toes, and total body. These areas correspond to typical patient complaints, such as “my arm hurts,” and include areas not included in the fibromyalgia criteria such as the head and genital regions. Those with total body pain were categorized separately rather than totaling the number of body parts affected. Central body parts included the head, neck, face, back/flanks, chest, abdomen, and buttocks/pelvis/groin and peripheral body parts included the arms, hands, legs and feet.

### Data analysis

The analyses were conducted using SAS software version 9.4 (Copyright© 2002–2012 by SAS Institute Inc., Cary, NC, USA). Demographic information and baseline characteristics were summarized by frequencies and percentages for categorical variables, and by median and interquartile range (IQR) for continuous variables. We re-defined painful body parts by merging some of the categories from registry data and derived a total of 13 total different categories (see above). We derived a variable reflecting the number of painful body areas for each subject based on these new categories and, given the continuous nature of this variable, we classified our sample into two groups using the median split method; subjects presenting with pain in < 5 body areas were categorized under the limited pain group, and those with ≥5 painful body areas were categorized under the diffuse pain group [19]. Unlike chronic widespread pain defined under 2010 ACR criteria for fibromyalgia, we defined diffuse pain in AMPS solely based on the number of painful body areas [13]. Differences in clinical and demographic characteristics, and psychological issues between these groups were assessed using Chi-squared or Fisher’s exact tests, as appropriate, for categorical variables and Wilcoxon Rank Sum Test for continuous variables.

## Results

**Fig. 1:**
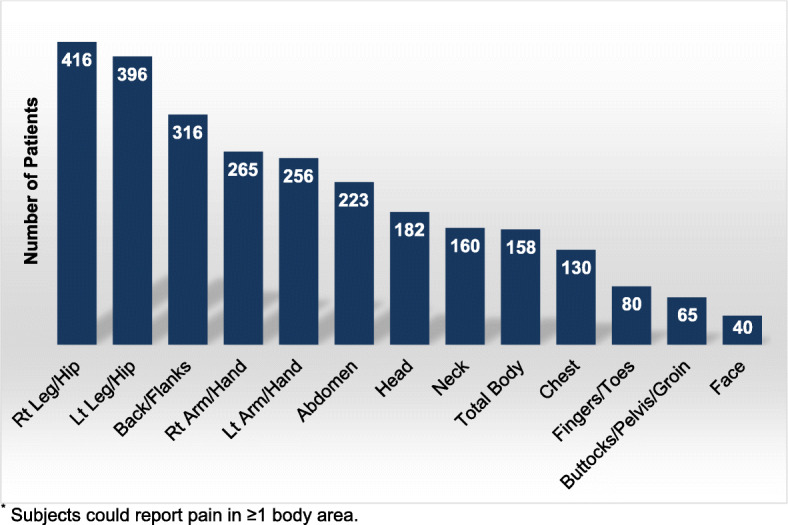
Different body parts reported as having pain^*^.^*^ Subjects could report pain in ≥1 body area

**Fig. 2:**
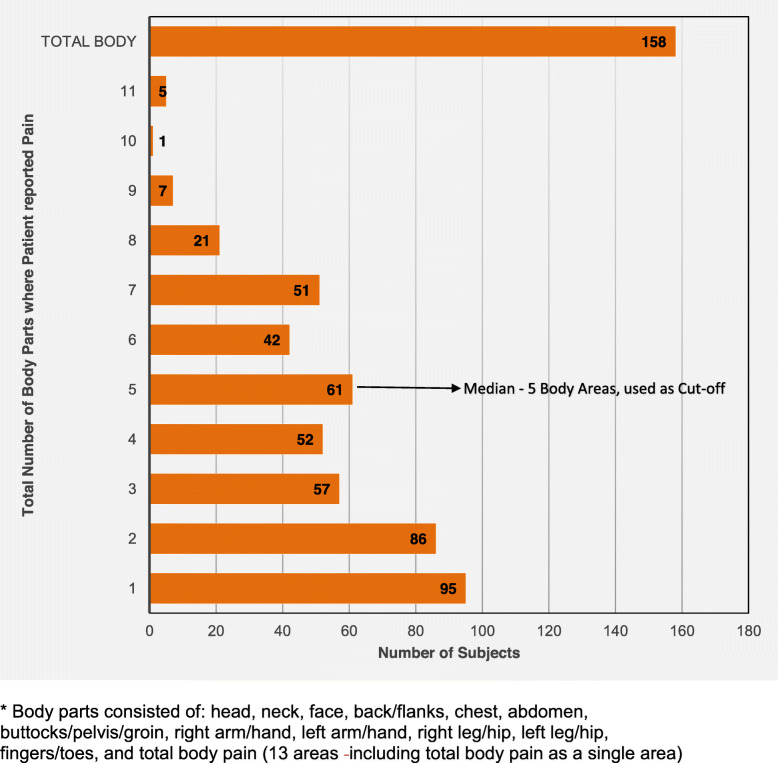
Number of painful body parts* reported (*N* = 636). * Body parts consisted of: head, neck, face, back/flanks, chest, abdomen, buttocks/pelvis/groin, right arm/hand, left arm/hand, right leg/hip, left leg/hip, fingers/toes, and total body pain (13 areas including total body pain as a single area)

There were a total of 636 subjects. The distribution and number of the 13 different designated areas where subjects reported having pain are shown in Figs. 1 & 2. The median number of painful areas was 5, which defined the median split between those with limited pain and those with diffuse pain (Fig. 2). Table 1 shows patient characteristics with the majority being Caucasian (79%), non-Hispanic (91%) and female (80%). The subjects with diffuse pain had a longer duration of pain (24 months vs. 12 months) so age at onset was similar to those with limited pain. Subjects with diffuse pain reported higher levels of pain, more disability and higher symptom severity scores and fewer trigger events (all *p-*values < 0.05, Table 1). The presence of at least one autonomic change at any given point was similar among the groups, both 14% (*p* = 0.94).

**Table 1:** Demographic, clinical and psychological characteristics based on pain type in subjects with amplified pain (*N* = 636)

Variables	All (*N* = 636)	Limited pain^*^ (*N* = 290)	Diffuse pain^*^ (*N* = 346)	*P-*value^†^
*Demographics, N (%)*				
Sex, female	509 (80%)	218 (75%)	291 (84%)	< 0.01^†^
Race				
Caucasian	502 (79%)	232 (80%)	270 (78%)	0.55
Black	53 (8%)	21 (7%)	32 (9%)	0.36
Other	78 (12%)	35 (12%)	43 (12%)	0.89
Ethnicity, non-Hispanic	581 (91%)	260 (90%)	321 (93%)	0.37
Age, median (IQR^|^)	14 (12–16)	14 (12–15)	15 (13–16)	< 0.001^†^
BMI, median (IQR)	22 (19–26)	21 (19–26)	19 (17–22)	0.56
Meet 2010 ACR Fibromyalgia Criteria^‡^	222 (35%)	23 (8%)	199 (58%)	< 0.0001^†^
*Patient Reported Outcome Measures, Median (IQR)*				
Current pain (0–10)	5 (3–7)	5 (2–7)	6 (4–7)	<.0001^†^
Most pain (0–10)	9 (8–10)	9 (8–10)	10 (8–10)	< 0.01^†^
Least pain (0–10)	3 (1–5)	3 (0–4)	4 (2–6)	<.0001^†^
Patient FDI (0–60)	22 (13–31)	19 (10–28)	25 (17–33)	<.0001^†^
Parent FDI (0–60)	22 (12–31)	18 (10–27)	25 (15–32)	<.0001^†^
WPI Score (scored 0–19)	6 (2–10)	2 (1–3)	10 (7–14)	<.0001^†^
SSS (scored 0–12)	6 (3–8)	4 (2–6)	7 (5–9)	<.0001^†^
Duration of symptoms (months)	18 (8–36)	12 (7–36)	24 (9–48)	< 0.01^†^
History of trigger event^^^	181 (28%)	98 (34%)	83 (24%)	< 0.01^†^
Attend Traditional School, yes [N, (%)]	344 (54%)	172 (59%)	172 (50%)	0.02^†^
Transient autonomic changes^§^, yes [N, (%)]	87 (14%)	40 (14%)	47 (14%)	0.94
*Self-reported Cognitive and/or Psychological Issues*^*¶*^*, N (%)*				
Anxiety / Panic attacks	288 (45%)	108 (37%)	180 (52%)	< 0.001^†^
Depression	173 (27%)	52 (18%)	121 (35%)	<.0001^†^
Obsessive Compulsive Disorder	55 (9%)	18 (6%)	37 (11%)	0.04^†^
Previous outpatient mental health care^║^	421 (66%)	177 (61%)	244 (71%)	0.01^†^
Previous psychiatric hospitalization	33 (5%)	7 (2%)	26 (8%)	< 0.01^†^
Suicide attempt	21 (3%)	6 (2%)	15 (4%)	0.12
Suicide ideation	117 (18%)	36 (12%)	81 (23%)	< 0.001^†^
*Adverse Childhood Experiences*^*¶*^*, N (%)*				
Household member Attempted/Committed Suicide	6 (1%)	1 (< 1%)	5 (2%)	0.23
Household member went to Prison	5 (1%)	2 (1%)	3 (1%)	1.00
Parents are Divorced/Separated	123 (19%)	45 (16%)	78 (23%)	0.03^†^
Parents or other household member with Alcohol/Drug problem	12 (2%)	3 (1%)	9 (3%)	0.24
Verbal/Physical/Sexual Abuse	7 (1%)	2 (1%)	5 (2%)	0.46
Other adverse childhood experiences	82 (13%)	39 (14%)	43 (12%)	0.70
*Number of Adverse Childhood Experiences, N (%)*				
0	445 (70%)	211 (73%)	234 (68%)	0.24
1	151 (24%)	67 (23%)	84 (24%)	
2	31 (5%)	10 (4%)	21 (6%)	
3	6 (1%)	2 (1%)	4 (1%)	
4	3 (< 1%)	0	3 (1%)	
*Type of Pain, N (%)*				
Constant	487 (77%)	216 (74%)	271 (78%)	0.04^†^
Intermittent	117 (18%)	61 (21%)	56 (16%)	
Constant and intermittent	26 (4%)	8 (3%)	18 (5%)	
No pain	6 (1%)	5 (2%)	1 (< 1%)	

Missing data: race (3), ethnicity (7), BMI (7), 2010 ACR fibromyalgia criteria (57), current pain (1), most pain (3), least pain (2), FDI patient (9), FDI parent (10), WPI (57), SSS (57), pain duration (1)

*Abbreviations*: *N* number of subjects, *IQR* Interquartile Range, *BMI* body mass index, *ACR* American College of Rheumatology, *FDI* Functional Disability Index, *WPI* Widespread Pain Index, *SSS* Symptom Severity Score

^*^Subjects with < 5 painful body regions categorized in Limited Pain and, subjects with ≥5 painful body regions categorized in Diffuse Pain

^†^*P*-value statistically significant if < 0.05

^‡^Based on 2010 American College of Rheumatology fibromyalgia criteria, subjects are categorized into two groups – meet the criteria if their WPI ≥ 7 and SSS ≥ 5 or WPI 3–6 and SSS ≥ 9, symptoms present for at least 3 months and absence of a disorder that explains the pain; and not meeting the criteria if not meet any of these conditions

^^^History of trigger event includes: major trauma, minor trauma, illness or surgery

^§^Autonomic changes categories: subjects could report or demonstrate an autonomic change (including temperature change, cyanosis, edema) in > 1 category

^║^Previous outpatient mental health care defined as seen at least once by a counselor/therapist/psychologist for pain

^¶^Subjects could report more than one

Our subject population included 330 (52%) outside our traditional catchment area. Of these, 143 (43%) had limited pain and 187 (57%) had diffuse pain. This is comparable to those inside our catchment area (147 (48%) with limited and 159 (52%) with diffuse pain).

**Fig. 3:**
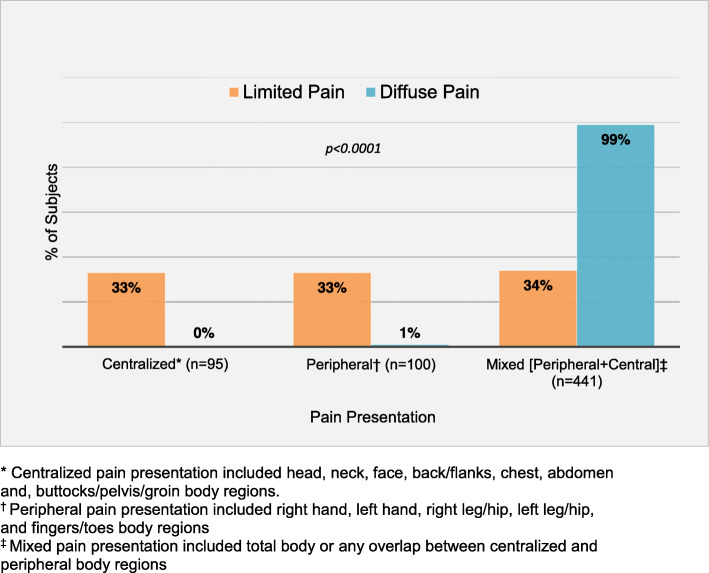
Pain presentation stratified by limited and diffuse pain (*N* = 636). * Centralized pain presentation included head, neck, face, back/flanks, chest, abdomen and, buttocks/pelvis/groin body regions. ^†^ Peripheral pain presentation included right hand, left hand, right leg/hip, left leg/hip, and fingers/toes body regions. ^‡^ Mixed pain presentation included total body or any overlap between centralized and peripheral body regions

Subjects with diffuse pain reported more psychologic symptoms, specifically anxiety (52% vs 37%; *p* < 0.001), depression (35% vs 18%; *p* < 0.001), and suicidal ideation (23% vs 12%; *p* < 0.001) and had more mental health therapy including outpatient mental health care (71% vs 61%; *p* = 0.01) and mental health hospitalization (8% vs 2%; *p* < 0.01) than those with localized pain. The presence of at least one ACE was reported among a quarter of the subjects and there was a slight increase in the incidence of parental divorce among those with diffuse pain (*p* = 0.03, Table 1). Subjects with diffuse pain almost always had both peripheral and central body pain but there was an even distribution between peripheral, central and mixed pain in those with limited pain (Fig. 3).

Criteria for adult fibromyalgia were fulfilled by 35% of the subjects, mostly those with diffuse pain (58% of those with diffuse pain) but 8% of those with limited pain also fulfilled these criteria (Table 1). The characteristics of those fulfilling adult fibromyalgia criteria are shown on Tables 1, 2 & 3. Notably, they had greater functional disability (FDI: 29 [IQR: 20–35] vs 19 [IQR: 10–27]; *p* < 0.0001), regardless of having widespread pain or limited pain (Tables 2 & 3).

**Table 2:** Demographics and pain related characteristics in population with amplified pain stratified by whether or not the groups meet American College of Rheumatology adult fibromyalgia criteria (1)(*N* = 579^*^)

Variables median (IQR)	Meet ACR fibromyalgia criteria^†^		*P*-value^‡^
	Yes (***N*** = 222)	No (***N*** = 357)	
Age	15 (14–16)	13 (11–15)	<.0001^‡^
Female sex	194 (87%)	270 (76%)	<.001^‡^
Number of Painful Body Parts	10 (6–12)	3 (2–5)	<.0001^‡^
Duration of pain (months)	24 (12–48)	12 (7–36)	<.001^‡^
Current pain (scored 0–10)	6 (4–8)	5 (2–7)	<.0001^‡^
Most pain (scored 0–10)	10 (9–10)	9 (8–10)	<.01^‡^
Least pain (scored 0–10)	4 (2–6)	3 (0–5)	<.0001^‡^
Patient FDI (scored 0–60)	29 (20–35)	19 (10–27)	<.0001^‡^
SSS (scored 0–12)	8 (6–10)	4 (2–6)	<.0001^‡^
WPI (scored 0–19)	11 (8–15)	2 (1–5)	<.0001^‡^

Missing data: current pain (1), most pain (2), least pain (2), FDI patient (9), FDI parent (10), pain duration (1)

*Abbreviations*: *N* number of subjects, *IQR* Interquartile Range, *FDI* Functional Disability Index, *WPI* Widespread Pain Index, *SSS* Symptom Severity Score

^*^Missing data on 2010 American College of Rheumatology fibromyalgia criteria on 57 subjects due to missing data on WPI and SSS

^†^Based on 2010 American College of Rheumatology fibromyalgia criteria, subjects are categorized into two groups – meet the criteria if their WPI ≥ 7 and SSS ≥ 5 or WPI 3–6 and SSS ≥ 9, symptoms present for at least 3 months and absence of a disorder that explains the pain; and not meeting the criteria if not meet any of these conditions

^‡^*P*-value statistically significant if < 0.05

**Table 3:** Difference in demographics and pain related characteristics in subjects grouped by diffuse versus limited pain and further stratified by fulfilment of American College of Rheumatology fibromyalgia criteria (*N* = 579*)

	Variables median (IQR)	Limited pain	Diffuse pain	*P-*value^‡^
Meeting 2010 ACR Criteria for Fibromyalgia^†^ (*N* = 222)	Denominator	23	199	
	Age	15 (14–16)	15 (14–16)	0.65
	Sex, Female n (%)	22 (96%)	172 (86%)	0.32
	Duration of pain (months)	35 (24–60)	24 (10–48)	0.27
	Current pain (0–10)	5 (3–6)	6 (4–8)	0.03^‡^
	Most pain (0–10)	9 (8–10)	10 (9–10)	0.53
	Least pain (0–10)	3 (1–5)	4 (2–6)	0.04^‡^
	Patient FDI (0–60)	29 (16–33)	29 (20–36)	0.26
	SSS	9 (7–10)	8 (6–10)	0.35
	WPI	7 (5–8)^†^	11 (9–16)	<.0001^‡^
Not Meeting 2010 ACR Criteria for Fibromyalgia^†^ (*N* = 357)	Denominator	244	113	
	Age	13 (11–15)	14 (12–15)	0.35
	Sex, Female n (%)	181 (74%)	89 (79%)	0.09
	Duration of Pain (months)	12 (6–36)	14 (7–36)	0.10
	Current pain (0–10)	5 (2–7)	5 (3–7)	0.19
	Most pain (0–10)	9 (8–10)	10 (8–10)	0.06
	Least pain (0–10)	3 (0–5)	3 (0–5)	0.13
	Patient FDI (0–60)	19 (10–27)	18 (11–27)	0.31
	SSS	4 (2–6)	4 (3–6)	0.16
	WPI	1 (1–3)	7 (4–9)	<.0001^‡^

Missing data: current pain (1), most pain (2), least pain (2), FDI patient (9), FDI parent (10)

*Abbreviations*: *N* number of subjects, *IQR* Interquartile Range, *FDI* Functional Disability Index, *WPI* Widespread Pain Index, *SSS* Symptom Severity Score

^*^Missing data on 2010 American College of Rheumatology fibromyalgia criteria on 57 subjects due to missing data on WPI and SSS

^†^Based on 2010 American College of Rheumatology fibromyalgia criteria, subjects are categorized into two groups – meet the criteria if their WPI ≥ 7 and SSS ≥ 5 or WPI 3–6 and SSS ≥ 9, symptoms present for at least 3 months and absence of a disorder that explains the pain; and not meeting the criteria if not meet any of these conditions

^‡^*P*-value statistically significant if < 0.05

Overall, 18% of all subjects reported exclusive intermittent pain. The incidence of intermittent pain was 21 in those with limited pain versus 16% in those with diffuse pain (*p* = 0.04). A small proportion had both constant pain and intermittent pains elsewhere in their body (3% in limited pain group vs 5% in diffuse pain group) (*p* = 0.04, Table 1). A few subjects (*N* = 6) had resolution of their amplified pain by the time of their initial visit to the AMPS clinic, 4 of whom had previously been diagnosed with amplified pain by a pediatric rheumatologist. However, based on Table 4, when comparing only the subjects who presented with constant pain versus intermittent pain (*N* = 604), we found no significant difference in distribution of subjects with intermittent pain based on limited versus diffuse pain (52% vs 48%, chi-square *p* = 0.13). However Apart from that, subjects with intermittent pain reported less widespread pain compared to those with constant pain (median WPI 4 [IQR: 1–9] vs. 6 [IQR: 2–11], respectively) and this was significant (*p* = 0.03) (Table 4). Similarly, those with intermittent pain reported less somatic complaints with a median SSS of 4 (IQR: 2–6) compared to a median score of 6 (IQR: 4–8) among those with constant pain (*p* < 0.0001). We observed a trend of less psychological problems reported by subjects with intermittent pain, however, only anxiety (34% in intermittent pain group vs 48% in constant pain) and suicidal ideation (12% in intermittent pain group vs 21% in constant pain) were found to be significantly different between the groups (*p* = 0.01 and *p* = 0.03, respectively, Table 4). Subjects with intermittent pain reported attending school more regularly (64%) than subjects with constant pain (51%) (*p* = 0.01). Overall, we observed lower symptom severity, pain and psychological co-morbidities in subjects with intermittent pain. There were no associations between intermittent pain and pain diagnosis given by the treating physician (Diffuse AMPS vs Localized AMPS, *p* = 0.46).

**Table 4:** Demographic, clinical and psychological characteristics based on intermittent versus constant pain among (*N* = 604*)

Variables median (IQR)	Constant pain (*N* = 487)	Intermittent pain (*N* = 117)	*P*-value^†^
Demographics and Patient Reported Outcome (PRO) Measures			
Age	14 (13–16)	13 (11–15)	< 0.001^Ɨ^
Sex, Female	89 (18%)	30 (26%)	0.07
Duration of pain (months)	18 (8–36)	24 (10–48)	0.04 ^Ɨ^
Current pain (0–10)	6 (4–8)	0 (0–2)	< 0.0001 ^Ɨ^
Most pain (0–10)	10 (9–10)	9 (8–10)	< 0.001 ^Ɨ^
Least pain (0–10)	4 (2–6)	0 (0–0)	< 0.0001 ^Ɨ^
Patient FDI (0–60)	25 (17–32)	13 (7–21)	< 0.0001 ^Ɨ^
SSS	6 (4–8)	4 (2–6)	< 0.0001 ^Ɨ^
Autonomic changes^§^, Yes	69 (14%)	12 (10%)	0.26
WPI	6 (2–11)	4 (1–9)	0.03 ^Ɨ^
History of trigger event	145 (30%)	25 (21%)	0.07
Attend School	248 (51%)	75 (64%)	0.01 ^Ɨ^
Patient Reported Psychological Problems^¶^			
Anxiety	234 (48%)	40 (34%)	0.01 ^Ɨ^
Depression	140 (29%)	26 (22%)	0.15
Suicidal Ideation	100 (21%)	14 (12%)	0.03 ^Ɨ^
Previous outpatient mental health care	332 (68%)	69 (59%)	0.06
Previous psychiatric hospitalization	30 (6%)	2 (2%)	0.06
Pain Diagnosis			
Diffuse AMPS	301 (62%)	68 (58%)	0.46
Limited AMPS	186 (38%)	49 (42%)	

*Abbreviations*: *N* number of subjects, *IQR* Interquartile Range, *FDI* Functional Disability Index, *WPI* Widespread Pain Index, *SSS* Symptom Severity Score

Missing data: current pain (1), most pain (3), least pain (1), FDI patient (8), FDI parent (10), WPI (53), SSS (53)

^*^We eliminated the 26 subjects who reported both constant and intermittent pain and 6 subjects with no pain

^†^*P*-value statistically significant if < 0.05

^¶^Subjects could report more than one

^§^Autonomic changes categories: subjects could report or demonstrate an autonomic change (including temperature change, cyanosis, edema) in > 1 category

## Discussion

The majority of a large population of children presenting for evaluation to a subspecialty pediatric rheumatology pain clinic had diffuse pain, but just over half met criteria for fibromyalgia according to the 2010 American College of Rheumatology criteria for adults [13]. Although Ting, et al., found the fibromyalgia criteria useful in adolescent girls to discriminate between those with fibromyalgia and localized chronic pain, this study was relatively small and did not include information on potential subjects who were excluded [20]. If juvenile fibromyalgia is a valid construct, it needs to be recognized as including only a relatively small subset of children with pain presenting to pediatric rheumatology clinics; we had a sizable number of children with localized pain who fulfilled adult fibromyalgia criteria. Studies limited to those fulfilling criteria for fibromyalgia miss out on a large segment of the population of children with diffuse amplified pain.

We hypothesized that those with limited pain would be more likely to have a history of a transient peripheral autonomic changes, but not enough to be classified as having CRPS, but this was not the case. It may be that children with transient peripheral autonomic changes should not be included in those with CRPS [11]. Those with limited pain were more likely to report prior trigger events, typically to the involved body part. However, only 34% of those with limited pain reported a trigger event compared to 54% of children with CRPS [21]. Further study is warranted.

Classifying children with amplified pain into either localized or diffuse involvement seems to be a valid construct since the latter displayed not only more severe pain and dysfunction, but also more psychological distress that needs to be recognized and addressed. The limited pain group had an overrepresentation of younger boys and a trend towards a higher incidence of preceding trauma. This group reported less dysfunction and fewer psychological symptoms. This may be, in part, due to a shorter duration of pain than those with diffuse pain (1 year vs 2 years on average). Alternatively, this earlier presentation may be due the more frequent history of trigger events and thereby an earlier recognition of the diagnosis.

The extent of limited pain is a matter of debate, however, it does not limit itself to the periphery, as typically does CRPS and, in our study, pain involved the core in two thirds of subjects. At times, it is limited to a single body area such as the abdomen, chest or head which leads to sub-specialty evaluations that are focused on organic causes and therapies. This may delay the diagnosis and treatment of amplified pain. We did not, however, assess the extent of prior evaluations between the groups or in those with limited pain of a specific body part such as abdomen. The number of body parts to define limited versus diffuse needs further study since the median split method may not be the best given the continuous nature of the data [19].

We identified a significant number of children, nearly a fifth of our population, who report intermittent pain, either localized or diffuse and a smaller subset of those with both constant pain in one area of their body and intermittent pains in another.. We hypothesized that those with intermittent pain would tend to have more limited pain but this was not the case as we found them distributed about equally within the limited vs diffuse pain groups (52% vs 48%).These children, like those with constant pain, need to be recognized and treated. It is our impression that those with intermittent pain do better in the long term but this needs formal investigation. However, this group did not have a longer duration of pain, as we hypothesized, but, reassuringly, they were more functional than those with constant pain. Additionally, they reported less anxiety and suicidal ideation than children with constant pain.

Forming validated, unified definitions of the various forms of amplified pain in the pediatric population is an important next step to help in the evaluation and treatment [22]. Additionally, the long-term outcomes including non-pain outcomes such as suicide, disordered eating, postural orthostatic tachycardia syndrome, substance abuse and other chronic pains (e.g., headache, functional abdominal pain) need to be included in such outcome studies.

The psychological burden is significant. It is our practice to routinely, formally integrate psychological assessments and care into our treatment of amplified pain. Suicidality was found in a significant proportion (18%) as was prior suicide attempt (3%). Subjects with diffuse pain had more suicidal ideation than those with limited amplified pain (23% vs 12%, *p* < 0.001) as did those with constant pain versus those with intermittent pain (21% vs 12%, *p* = 0.03). There may be intrinsic differences in children who develop diffuse pain compared to those with more limited pain or it may be the increased pain duration, severity and associated dysfunction that leads to more mental health issues. Children with diffuse pain and potential suicidality should be monitored closely with appropriate safety plans.

Children in this study also experienced other psychiatric co-morbidities, especially anxiety (45%) and depression (27%). Both were more frequent in those with diffuse pain, as well as prior mental health therapy, including hospitalization. These findings support our hypothesis that diffuse amplified pain incurs a greater psychologic burden. These diagnoses were self-reported but we suspect that, given the social stigma associated with mental health, psychological issues were underreported which may bias our findings. Likewise, we know of several children who, years later, reported they had been victims of abuse. The role of adverse childhood experiences is yet to be fully understood, but this may be an important factor in the development of amplified pain [23]. In particular, parental divorce, in our study, seems to be more common in those with diffuse pain. The actual burden of psychiatric illness in this patient population is likely much greater than the initial reports suggest.

Our study has several limitations. First, this is a quaternary academic center and there may have been significant referral bias as less severe and less complex patients may have been managed successfully by primary care physicians or other pain centers. Therefore, our findings may not be generalizable and may overestimate the prevalence of those with more severe dysfunction and mental health issues including suicidality. Nonetheless, our study demonstrates the importance of routine psychological counseling for these children and that psychological co-morbidities pose a real risk. Much of our data were patient reported. We did not have independent confirmation of anxiety, depression and other psychologic variables such as adverse childhood experiences. Additionally, data were taken at the time of the initial visit, and we know of many children who will manifest such symptoms, develop suicidal ideation, or report adverse childhood experiences much later. Long term acquisition of these data are needed. Another limitation is missing data, however, the missing data were specific to psychological variables and secondary pain diagnosis. Even with the missing data, our study provides the largest cohort of children with AMPS.

## Conclusions

Children and adolescents with AMPS have a spectrum of presenting manifestations, including limited and diffuse pain, many of whom do not fulfill the diagnostic criteria for fibromyalgia. This should be taken into account regarding pediatric studies of children diagnosed with fibromyalgia. The pain of limited amplified pain can be peripheral, central or both and most children with diffuse pain have pain both centrally and peripherally. Those with diffuse pain have a longer duration of pain prior to presentation, more disability and greater symptomatology and barely more than half fulfill criteria for fibromyalgia. Those with diffuse pain reported more psychological symptoms including anxiety, depression, use of mental health and suicidal ideation. There is a sizeable number of children who report intermittent pain that should be recognized as part of the amplified pain spectrum. Further studies need to determine if the initial pain presentation (limited vs diffuse, constant vs intermittent) affects treatment outcomes and long range morbidity and elucidate the risk factors for the transition from localized to diffuse pain.

## Abbreviations

ACE: Adverse childhood experiences
ACR: American College of Rheumatology
AMPS: Amplified musculoskeletal pain syndrome
CRPS: Complex regional pain syndrome
FDI: Functional disability inventory
IQR: Interquartile range
IRB: Institutional Review Board
PRO: Patient reported outcomes
REDCap: Research Electronic Data Capture
SSS: Symptom severity score
WPI: Widespread pain index
